# Current Options for Second-Line Systemic Therapy in Metastatic Renal Cell Carcinoma

**DOI:** 10.15586/jkcvhl.v9i3.243

**Published:** 2022-09-29

**Authors:** Iraklis C. Mitsogiannis, Maria Mitsogianni, Maria Papathanassiou, Maria Anagnostou, Ioannis Tamposis, Lampros Mitrakas, Maria Samara, Vassilios Tzortzis, Panagiotis J. Vlachostergios

**Affiliations:** 1 Second Department of Urology, National and Kapodistrian University of Athens, Sismanoglio General Hospital, Athens, Greece;; 2 Fourth Department of Internal Medicine, “Hygeia” Hospital, Athens, Greece;; 3 Department of Pathology, University of Thessaly, Faculty of Medicine, University Hospital of Larissa, Larissa, Greece;; 4 Department of Computer Science and Biomedical Informatics, University of Thessaly, Lamia, Greece;; 5 Department of Urology, University of Thessaly, Faculty of Medicine, University Hospital of Larissa, Larissa, Greece;; 6 Division of Hematology and Medical Oncology, Department of Medicine, Weill Cornell Medicine, New York, NY, USA

**Keywords:** angiogenesis, immunotherapy, renal cell carcinoma, second line, tyrosine kinase inhibitor

## Abstract

Standard systemic therapy of advanced renal cell carcinoma (RCC) involves targeting angiogenesis, mainly through tyrosine kinase inhibitors (TKI) against the vascular endothelial growth factor receptor (VEGFR) pathway and targeting the immune checkpoints, namely, programmed death-1 (PD-1) or its ligand (PD-L1), and cytotoxic T-lymphocyte-associated protein 4 (CTLA4). With current strategies of combining these two approaches in the front-line setting, less is known about optimal selection of therapy upon development of resistance in the second and later lines of treatment for progressive disease. This review discusses currently available therapeutic options in patients who have progressive RCC after prior treatment with double immune check-point inhibitors (ICIs) or ICI-TKI combinations.

## Introduction

Renal cell carcinoma (RCC) is the second most common malignancy of the urinary system and accounts for 4–5% of yearly estimated new cancer cases in the United States (1, 2). Approximately 80% of cases are histologically classified as clear-cell renal cell carcinoma (ccRCC). Advances in diagnostic imaging have led to earlier diagnosis of renal tumors in the last decades (3); nonetheless, 16% of patients initially present with metastatic disease, while recurrences of early stage disease are not uncommon (2, 4). Chemotherapy has shown disappointing results and currently has no place in the treatment of ccRCC (5). The introduction of novel treatments such as immune check-point inhibitors (ICIs) and targeted agents in the first and subsequent treatment lines has substantially improved prognosis for metastatic RCC. However, the reported 5-year survival rates remain as low as 14% (2).

Combined first-line approaches of immunotherapy ICIs and tyrosine kinase inhibitors (TKIs) targeting tumor angiogenesis through vascular endothelial growth factor (VEGF) receptor signaling include pembrolizumab plus lenvatinib, pembrolizumab plus axitinib, and nivolumab plus cabozantinib; these treatment options have demonstrated survival benefit in all treatment-naïve patient subgroups (6–8), whereas double ICIs with nivolumab plus ipilimumab is a valid first-line option for patients with intermediate- and poor-risk disease (9). Single-agent immunotherapy or TKI can be considered in select cases (10). Despite good initial response rates, acquired resistance occurs almost universally. Optimal post-progression treatment sequencing represents a clinical challenge and is highly individualized, depending, to a great extent, on previous regimens, disease burden, biological tumor behavior, and patient’s medical history and health status.

This review discusses currently available therapeutic options in patients who have progressive RCC after prior treatment with double ICIs or ICI-TKI combinations.

## Second-Line Anti-Angiogenic Therapies

Treatment with an anti-VEGFR (Vascular Endothelial Growth Factor Receptor), TKI or mTOR inhibitor should be considered for patients progressing on IO or IO-TKI combination and vice versa, although head-to-head comparisons of different agents are lacking and current data to support this approach are based on small studies (11).

### Sunitinib

Sunitinib is a multi-TKI inhibitor and was the first targeted agent to be approved in metastatic ccRCC, initially by demonstrating efficacy in cytokine-refractory disease and soon thereafter in treatment-naïve patients when compared to interferon-alpha (12). In recent years, sunitinib has lost ground as the preferred modality in the first-line setting based on the findings of numerous phase III studies of ICI-TKI and ICI-ICI combinations that outperformed it as a comparator arm (11). There are sparse data on the role of sunitinib as a second-line therapy, mostly after prior TKI or cytokine-based therapy and less frequently after ICIs. The RECORD-3 phase II trial demonstrated activity of the everolimus-sunitinib sequence with a median combined progression-free survival (PFS) of 21.7 months and a median OS of 22.4 months, which however was inferior compared to the reverse sequence (sunitinib-everolimus) (13). Rechallenge with sunitinib as third-line treatment and beyond has also shown efficacy supported by a median PFS of 7.9 months (14). In the ICI-based frontline therapy era, prospective evaluation of sunitinib as a second-line agent in the INMUNOSUN-SOGUG phase II trial has yielded a median PFS of 5.6 months and OS of 23.5 months (15). Importantly, those patients who demonstrated better responses to first-line ICI-based therapies were also the ones who benefited the most from sunitinib (15). A larger, real-world retrospective study of 102 patients confirmed the activity of second-line sunitinib following immunotherapy, showing an objective response rate (ORR) of 22.5%, a median time to treatment discontinuation of 5.4 months, and a median OS of 15.6 months (16).

### Pazopanib

Pazopanib is a VEGFR-, PDGFR-, and KIT-receptor inhibitor, which is clinically active in metastatic ccRCC, according to the results of a phase III study that included 46% of cytokine-pretreated patients (17). Pazopanib resulted in a longer median PFS compared to placebo (7.4 vs. 4.2 months; HR 0.54; 95% CI 0.35–0.84; P < 0.001) in this pretreated population. However, no survival benefit was shown possibly because of the high crossover rate. Pazopanib is noninferior to sunitinib, in terms of PFS and OS, according to the results of a phase 3 trial in treatment-naïve patients (18); furthermore, the drug has a more favorable toxicity profile and seems to be better tolerated by the majority of patients (19).

Pazopanib was demonstrated to be efficacious in a phase 2 trial, which included patients pretreated with sunitinib or bevacizumab; median PFS and 24-month OS were 7.5 months (95% CI 5.4–9.4 months) and 43%, respectively, regardless of the previously used agent (20). Another single-arm phase 2 study confirmed the efficacy of pazopanib after TKI-failure in cabozantinib or nivolumab noneligible patients (median PFS 6.7 months (95% CI 3.7–11.2), median OS 20.6 months (95% CI 12.6–27.4)) (21). A meta-analysis of six studies further supported the use of pazopanib in noncytokine pretreated patients, despite the lack of robust prospective/randomized data (22). In the post-frontline immunotherapy era, real-world evidence of second-line pazopanib support its safety and efficacy as shown by a median PFS of 13.5 months and a 12-month OS rate of 89% (23).

### Sorafenib

Sorafenib is a multi-VEGFR inhibitor and one of the first pharmacologic agents used as targeted therapy in metastatic ccRCC. A phase III trial (24) comparing sorafenib to placebo in pretreated patients reported significantly longer median PFS (5.5 vs. 2.8 months; HR 0.44; 95% CI 0.35–0.55, P = 0.000001). OS was similar, most likely due to crossover. Indirect comparison indicates superiority of cabozantinib, nivolumab, and everolimus over sorafenib as second-line treatment (25). Sorafenib is nowadays reserved for later treatment lines or when other options are not available.

### Axitinib

Axitinib is a potent inhibitor of VEGFR-1, -2, and -3. In the landmark phase 3 AXIS trial (26, 27), axitinib was compared to sorafenib as the second line treatment of metastatic ccRCC. Median PFS was longer with axitinib (8.3 vs. 5.7 months, hazard ratio (HR) 0.66, 95% confidence interval (CI) 0.55–0.78, one-sided P < 0.0001). Superiority of axitinib was particularly evident in cases of prior cytokine treatment (PFS 12.1 vs. 6.5 months; HR 0.46, 95% CI 0.32–0.68), compared to prior sunitinib (PFS 4.8 vs. 3.4 months; HR 0.74, 95% CI 0.57–0.96). Overall survival (OS) did not differ significantly between the two groups (20.1 vs. 19.2 months, HR 0.70, 95% CI 0.80–1.17; one-sided P = 0.374) (27). Patient-reported kidney-specific symptoms and health status, measured by the Functional Assessment of Cancer Therapy (FACT) Kidney Cancer Symptom Index (FKSI) and the European Quality of Life self-report questionnaire (EQ-5D) in the AXIS trial were similar between axitinib and sorafenib, with substantial worsening at the end of treatment mainly due to disease progression (28). Longer duration of first-line treatment (cytokines or sunitinib) was positively associated with survival in a post hoc analysis (29). Common adverse events included diarrhea and hypertension, while toxicity led to treatment discontinuation in 4% of patients. Interestingly, high (>90 mmHg) diastolic blood pressure was associated with prolonged overall survival (27).

Prognostic factors associated with better response to axitinib compared to sorafenib, in the sunitinib-pretreated group of patients, include nonbulky, favorable-, or intermediate-risk disease, as well as the absence of liver and bone metastases (30). The efficacy of axitinib in favorable risk sunitinib-pretreated patients was further confirmed in a single-arm, phase 2 study of 21 patients, which reported responses in 33% of patients and a median PFS of 17 months (95% CI 14–20) (31).

Axitinib after immune-checkpoint inhibition has also been prospectively evaluated in a single-arm phase 2 study (32). A total of 40 patients were included, the majority of which (63%) were treated with nivolumab directly before trial enrollment. ICI combination with nivolumab and ipilimumab was the most recently received regimen in 15% of the patients. An ORR of 45% and a median PFS of 8.8 months (95% CI 5.7–16.6) were noticed (32).

Quality-of-life (QOL) analysis using the Medical Outcomes Study 36-Item Short Form in a real-world clinical practice study of 124 patients showed that all eight QOL scores after the introduction of axitinib were superior to those before its introduction, and there were significant differences in two of the eight scale scores between surveys conducted before and 12 weeks after the introduction of axitinib (33).

### Cabozantinib

Cabozantinib affects cancer growth by inhibiting VEGFR-2 and other tyrosine kinases, such as MET and AXL, which are associated with resistance to sunitinib and poor prognosis (34). It has been established as a subsequent treatment option of metastatic ccRCC based on the results of the phase 3 METEOR trial (35). Patients progressing on ≥1 VEGFR-TKI were randomized to either cabozantinib or the mTOR inhibitor everolimus. Cabozantinib demonstrated both PFS (7.4 vs. 3.9 months, HR 0.51, 95% CI 0.41–0.62, P < 0.0001) and OS benefit (21.4 vs. 17.1 months, HR 0.71, 95% CI 0.58–0.85, P = 0.002). In subgroup analysis, benefit was maintained irrespective of prior use of ICI. A phase 2 trial evaluating cabozantinib after progression on the current first-line standard-of-care (predefined cohorts of ICI-ICI and anti-VEFGR/ICI combination), is ongoing (36).

### Tivozanib

The VEGFR-inhibitor tivozanib is the most recently approved TKI in the treatment of relapsed RCC. It has been evaluated in the randomized phase III TIVO-3 trial, where it was compared to sorafenib after disease progression on at least two treatment lines (37, 38). Clear-cell histology accounted for more than 95% of the cases in both arms. Tivozanib led to an improvement of PFS (6 vs. 4 months, HR 0.73, 95% CI 0.56–0.94) but not of OS (16 vs. 19 months, HR 0.97, 95% CI 0.75–1.24) (38). Benefit was seen in patients who had previously received checkpoint inhibition and VEGFR TKIs and two different VEGFR TKIs (38).

### Lenvatinib

The VEGFR-targeting TKI lenvatinib in combination with the mTOR inhibitor everolimus has demonstrated PFS benefit compared to everolimus alone, according to the results of a phase 2 trial (14.6 vs. 5.5 months, HR 0.40, 95% CI 0.24–0.68) (39). It has been approved after failure of antiangiogenic treatment. The approved dose of lenvatinib is 18 mg. It has been tested whether a starting dose of 14 mg is noninferior in a randomized phase 2 trial, but both PFS and OS were numerically better in the 18 mg arm (40). Real world data indicates effectiveness of the Lenvatinib-everolimus combination after prior TKI with a median PFS of 6.4 (95% CI 4.1–10.8), and after immunotherapy with median PFS of 5.7 months (95% CI 4.1–10.5) and median OS of 14.8 months (95% CI, 10.2–23.9), as well as in heavily pretreated patients after a median of 3 previous treatment lines (41).

All available anti-angiogenic agents studied so far in the second line are summarized in Table 1.

**Table 1:** Randomized trials of second-line agents after VEGFR TKI monotherapy.

	Axitinib	Cabozantinib	Lenvatinib/Everolimus	Nivolumab
Study	Rini et al. (26)	Choueiri et al. (35)	Motzer et al. (39)	Motzer et al. (42)
MSKCC risk: favorable/int/poor	28/37/33	45/42/12	24/37/39	35/49/16
Comparator	Sorafenib	Everolimus	Everolimus	Everolimus
ORR, %	19	17	35	22
PD, %	22	12	4	35
PFS, mo	4.8	7.4	12.8	4.6
OS, mo	20.1	21.4	25.5	25.0

*Abbreviations*: VEGFR, vascular endothelial growth factor receptor; TKI, tyrosine kinase inhibitors; MSKCC, Memorial Sloan-Kettering Cancer Center; int, intermediate; ORR, objective response rate; PD, progression of disease; PFS, progression-free survival; OS, overall survival; mo, months.

## Second-Line Immunotherapy

### Nivolumab and Ipilimumab

Nivolumab monotherapy is indicated in patients who have progressed on anti-VEGR treatment, based on the results of the CheckMate 025 randomized phase 3 trial (42, 43). Nivolumab was compared to everolimus, and it demonstrated superiority in terms of ORR (23 vs. 4%), 5-year PFS (5 vs. 1%, HR 0.84, 95% CI 0.72–0.99), and OS (25.8 vs. 19.7 months, HR 0.73, 95% CI 0.62–0.85), although median PFS did not differ. Quality of life was also improved in the nivolumab group (42, 43). There are also data indicating the benefits of nivolumab post-progression in patients experiencing clinical benefit (44). Nivolumab after disease progression led to >30% reduction in tumor burden in 13% of the patients who stayed on treatment (44).

The combination of nivolumab and ipilimumab is a valid option for patients who are in good shape and did not receive it as first-line treatment. Salvage therapy with nivolumab and ipilimumab has demonstrated efficacy after progression on anti PD-1 targeted therapy in a small retrospective analysis of 44 patients. ORR was 20% and median was PFS 4 months (45). In another ambispective multicenter study with 45 mRCC patients rechallenged with nivolumab±ipilimumab, ORR was 16% (n = 7) for second-line ICI (45). Median PFS was 3.5 months, and median OS was 24 months (46). Factors associated with poorer PFS were a high number of metastatic sites, presence of liver metastases, and the use of an intervening treatment between ICI regimens, Eastern Cooperative Oncology Group performance status ≥2, and poor International Metastatic RCC Database Consortium score upon second-line ICI initiation (46). Conversely, a PFS longer than 6 months at first-line ICI was associated with better PFS during second-line ICI (46).

TiNivo-2 is a phase III randomized trial comparing nivolumab plus tivozanib to tivozanib monotherapy in metastatic RCC patients who have progressed following one or two lines of therapy including an ICI, with primary endpoint being PFS (NCT04987203).

### Pembrolizumab

Pembrolizumab as later-line treatment after failure of ICI has been evaluated in combination with lenvatinib in a phase 1b/2 trial with a total of 104 patients, the majority of which had received at least two treatment lines. The investigators described an ORR of 56%, and median DOR of 12.5 months (47).

### Atezolizumab

The combination of atezolizumab and bevacizumab demonstrated activity in patients progressing after atezolizumab or sunitinib. In this randomized phase 2 trial, 103 patients started second-line atezolizumab plus bevacizumab, of whom 44 had previously received atezolizumab as first-line monotherapy while 59 were previously treated with sunitinib (48). ORR was reported as high as 27%. The median PFS from the start of second line was 9 months. The median event follow-up duration was 19 months among the 25 patients without a PFS event (48). Atezolizumab combined with cabozantinib is currently being tested in the pivotal, global phase III CONTACT-03 trial in patients with inoperable, locally advanced or metastatic RCC who progressed during or following treatment with an ICI (NCT04338269).

All available ICI options studied so far in the second line are summarized in Table 2.

**Table 2:** Prospective and retrospective trials of second-line agents after ICI/VEGF inhibitor or ICI ± ICI.

	Ipilimumab/Nivolumab	Ipilimumab/Nivolumab	Lenvatinib/Pembrolizumab	Atezolizumab/Bevazicumab
Study	Gul et al. (45)	Vauchier et al. (46)	Lee et al. (47)	Powles et al. (48)
IMDC or MDACC risk: favorable/int/poor	20/64/7	23/25/53	17/59/24	21/76/3
ORR, %	20	16	62.5	27
PD, %	62	67	57	32
PFS, mo	4	3.5	12.2	8.7
OS, mo	NR	24	72% at 16 mo	NR


*Abbreviations*: VEGFR, vascular endothelial growth factor receptor; ICI, immune check-point inhibitors; IMDC, International Metastatic RCC Database Consortium; MDACC, MD Anderson Cancer Center; int, intermediate; ORR, objective response rate; PD, progression of disease; PFS, progression-free survival; OS, overall survival; mo, months; NR, not reported.

## Comparisons between Therapeutic Options in the Second-Line Setting

Several targeted anti-angiogenic agents used as monotherapy have been proved to be more efficient than sunitinib and have further reduced its clinical use. Cabozantinib led to superior ORR (20 vs. 9%) and PFS (8.6 vs. 5.3 months, HR 0.48, 95% CI 0.31–0.74) in treatment-naïve patients with intermediate- and high-risk disease (49).

With regard to treatment sequencing, sorafenib followed by sunitinib on disease progression appears to be equally efficient to sunitinib followed by sorafenib according to randomized, phase 3 data (50). On the other hand, everolimus followed by sunitinib has failed to demonstrate non-inferiority compared to sunitinib followed by everolimus in a randomized, phase 2 trial (51).

Axitinib does not seem to be superior to nivolumab as second-line option after targeted therapy, although a trend towards improved PFS was reported in a small retrospective study (52). Axitinib re-challenge as fourth- or later-line treatment has demonstrated clinical benefit in a recent case series (53).

According to a meta-analysis including patients progressing on VEGFR inhibition (20), cabozantinib was associated with a lower HR for disease progression and death compared to axitinib, everolimus, nivolumab, sorafenib, and best supportive care. It is, therefore, being currently recommended as a preferred second-line option in the latest NCCN guidelines (54). Retrospective real-world data further confirm PFS superiority of cabozantinib vs. nivolumab for ccRCC (7.8 vs. 5.4 months, P = 0.026) and good-risk disease (12.3 vs. 5.7 months, P = 0.022) (55).

## Controversies on Retreatment with Immune Checkpoint Inhibitors

From a biological standpoint, there is evidence of continued activity of anti-PD-1 blockade up to 3 months after initial administration and up to 9 months after at least three treatment cycles (56). Furthermore, because the mechanism of action of ICIs is mainly based on their ability to trigger adaptive immune response by reversing the inhibition and suppression of T cells in the tumor microenvironment, whether further continuation of ICI after reactivation of immune response is established is questionable (57). On the other hand, when compared with nonstop treatment with nivolumab for over 1 year, retreatment at progression in the CheckMate-153 trial resulted in inferior PFS and OS outcomes (58).

Even without interruption, patients treated with ICIs eventually experience progression of their disease due to the development of acquired resistance. Resistance mechanisms may involve insufficient generation of anti-tumor T cells, inadequate function of tumor-specific T cells, or impaired generation of memory T-cells (59, 60).

In the absence of predictive biomarkers, clinical phenotypic characteristics could offer some hints to guide the choice and sequencing of ICIs in ICI-pretreated patients. For example, treatment with the same ICI beyond progression might be an option for a subset of patients who experienced slow progression after initial response. On the other hand, patients with primary resistance to ICI as well as those with visceral disease or rapid deterioration might not benefit from this strategy but should rather be offered a course of TKI with faster onset of activity (61). After eliminating the most aggressive cancer clones, altering the tumor microenvironment, removing immunosuppressive signals, and releasing novel tumor-associated antigens, retreatment with a different ICI might be effective (62, 63). Combining ICIs against different immune targets might be one way to prevent or/and overcome resistance and improve outcomes in these patients.

## Hypoxia-Inducible Factor 2α Inhibition

Belzutifan is a potent, selective, oral small-molecule HIF-2α inhibitor with antitumor activity in clear-cell RCC (64). The drug at 120-mg dose was shown to be well tolerated and demonstrated significant activity translating into an ORR of 25% and a DCR of 80% in 55 patients with advanced ccRCC previously treated with at least one prior line of therapy (65, 66). While belzutifan is already FDA-approved for patients with von Hippel-Lindau (VHL) disease who require therapy for associated RCC, based on a phase 2, open-label, single-group trial (67), a phase 3 study comparing the drug with everolimus in advanced RCC after PD-1/PD-L1 therapy and TKI is underway (NCT04195750). There is also encouraging evidence of activity and tolerability of belzutifan in combination with cabozantinib in 53 previously treated patients, including immunotherapy and TKIs (cohort 2) (68). The study reported a confirmed ORR of 22% while the disease control rate (CR + PR + SD) was 92.7% and median PFS was 16.8 months (66).

## Local Therapies for Oligoprogressive Disease

Cytoreductive nephrectomy and metastasis-directed therapies involving resection, radiation, or ablation, either upfront or after initiation of systemic therapies, continue to have a role in the management of metastatic RCC (67). Their contribution in improving outcomes of patients with oligoprogressive disease after initial systemic therapy is less clear and requires even more careful patient selection due to lack of predictive biomarkers (68). Retrospective data suggest that patients with a Memorial Sloan-Kettering Cancer Center (MSKCC) good risk score or bone metastases may experience longer post-first oligoprogression OS compared to patients of the intermediate risk group (39 vs 29 months) or visceral metastases (not reached vs 31 months), respectively (69). Retroperitoneal lymph node dissection (RPLND) and metastasectomy may be beneficial when technically feasible in oligometastatic disease and a proportion of those may achieve long-term OS with aggressive resection (70). However, postoperative complications should be carefully weighed upon decision-making, particularly in hepatic resections which portend the highest risk for major ones (Clavien III-IV) in up to one-fourth of the patients (71).

Intraoperative cryotherapy may have a role in osseous metastases in controlling the metastatic tumor bed and offering long-term pain relief; however, more data are needed (72). Percutaneous multisite cryoablation may address various anatomic sites under ultrasound- or CT-guidance and is cost-effective further to having only a 2% rate of complications or local recurrence (73). Microwave ablation is also safe with a 15% rate of complications and durable local control in 93% of cases, although retrospective nature and small study size remains a limitation (74).

The most well-studied, noninvasive local approach is radiotherapy, mostly in the form of stereotactic body radiation (SBR). A large amount of prospective and retrospective studies and meta-analyses support the activity of this approach for treating oligometastatic disease in combination with TKIs or ICIs (75). Local control rates usually exceed 90%, even for intracranial disease, with severe toxicity being uncommon (<10%) (75). Although prospective evaluation of SBR plus nivolumab did not add any systemic benefit with respect to ORR (17%) and median OS (20 months) compared to historical controls of nivolumab alone in the NIVES study, more prospective trials such as the RAPPORT study are further investigating this with molecular correlates to provide insights into the underlying biology of combination SBR and ICI therapy (76, 77). With respect to clinical correlates, a good ECOG PS ≤1 predicts a longer OS benefit in such patients (78). Other retrospective studies suggest a greater benefit from SBR in patients with metachronous metastases and in those with smaller lesion size (<14 mm) (79, 80). Complete SBR (as opposed to incomplete SBR) may lead to improved cancer-specific survival in younger patients (age <55 years), with clear-cell tumors and low metastatic of <3 lesions (81). Another outcome often underscored in various studies is time to next systemic therapy initiation or time to systemic therapy escalation, which can be significantly prolonged with SBR for over a year, as well as with classic external beam radiation (82–88). Alternatively, SBR can significantly extend the duration of ongoing systemic therapy by more than 6 months without compromising the quality of life (89). Stereotactic radiosurgery (SRS) can achieve such good local control that the median OS rates after switching systemic therapy versus continuing the same are similar (24 vs 27 months) (90).

## Next-Line Treatment Selection

**Figure 1:**
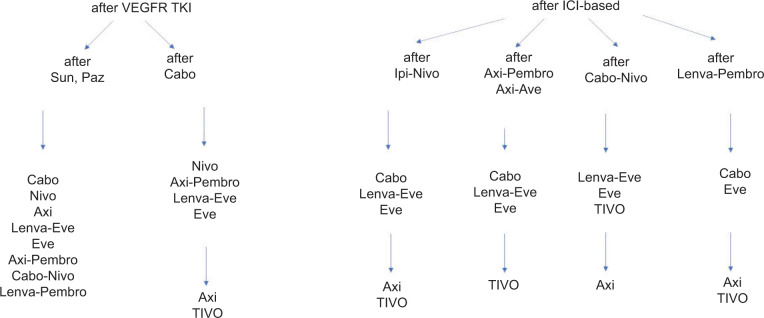
Suggested sequences for second- and third-line treatment of clear-cell RCC. *Abbreviations:* RCC, renal cell carcinoma; VEGFR, vascular endothelial growth factor receptor; TKI: tyrosine kinase inhibitor; ICI, immune checkpoint inhibitor; Sun: sunitinib; Paz: pazopanib; Cabo, cabozantinib; Nivo, nivolumab; Ipi: ipilimumab; Pembro, pembrolizumab; Axi, axitinib; Tivo, tivozanib; Lenva, Lenvatinib; Eve, everolimus.

Overall, there are no randomized comparisons between different treatment options for second and subsequent lines of therapy. Based on variable ORR, duration of responses, and PFS in individual studies, some general principles could be proposed. If long-term disease control was previously achieved on first-line single-agent VEGF inhibitor (i.e., pazopanib), patients could either continue VEGF inhibition alone with cabozantinib or axitinib or tivozanib, or in combination with an mTOR inhibitor (lenvatinib and everolimus), or receive ICI monotherapy with nivolumab. Besides, these options are supported by category 1 evidence according to current NCCN treatment guidelines; active second-line regimens include other TKIs (sunitinib, pazopanib), ICI-TKI combinations (axitinib-pembrolizumab, cabozantinib-nivolumab, lenvatinib-pembrolizumab, axitinib-avelumab), or double ICI combination (ipilimumab-nivolumab) (54). After primary or acquired progression on VEGF and ICI combination, non-ICI VEGF inhibition strategies with a different VEFGR-targeted agent should be implemented. According to ESMO guidelines, this strategy is associated with modest response rates and should be considered the standard of care (11). In the absence of strong evidence to support continued ICIs after progression on first-line ICI-based therapy, if disease control was achieved on ipilimumab plus nivolumab prior to progression, consideration could be given to VEGF and ICI combinations for second line, particularly lenvatinib plus pembrolizumab. These same options can be considered for third line and beyond, depending on what the patient has previously received. Proposed treatment algorisms for second- and third-line treatment of ccRCC are illustrated in Figure 1. Until present, therapeutic decisions in this setting are mostly guided by strength of evidence for each drug, its toxicity profile, disease aggressiveness, mechanism of action, and pattern of responses during prior lines of therapy, patient comorbidities, availability of clinical trials, patient and physician preferences, and financial concerns.

## Nonclear-Cell RCC

In this minority of clinicopathological RCC subtypes where phase III randomized studies are lacking, preferred frontline therapy consists of a VEGFR TKI, usually either cabozantinib or sunitinib, particularly in papillary tumors, although outcomes are usually inferior compared to ccRCC (11, 54, 91). Another alternative is the combination of lenvatinib and everolimus supported by phase 2 data (92). As a result, subsequent lines of therapy after progression may include either a different TKI or/and an ICI. The combination of cabozantinib and nivolumab demonstrated promising activity in the unclassified/papillary/translocation-associated cohort of a phase II study with a median ORR of 47% and OS of 28 months (93). One third of those patients had received one prior line of therapy with a VEGF or mTOR inhibitor (93). Nivolumab monotherapy in the phase IIIb/IV CheckMate 374 study in previously treated non-ccRCC patients also demonstrated clinically meaningful activity with median ORR of 13% and OS of 16 months (94).

Newer studies, mostly retrospective, have demonstrated improved OS with ICI-based frontline therapy compared to VEGF and mTOR-targeted agents (26 vs 16 vs 12 months, respectively) (95). Safety and efficacy results from the advanced non-ccRCC cohort of the phase IIIb/IV CheckMate 920 study confirmed the activity of first-line treatment with nivolumab and ipilimumab with a median ORR of 19% and OS of 21 months (96). Likewise, pembrolizumab monotherapy in the phase II KEYNOTE-427 study resulted in high response rates of 28.8% for papillary, 9.5% for chromophobe, and 30.8% for unclassified tumors (97). Thus, it is likely that the future landscape of second and subsequent-lines of treatment in non-ccRCC patients may face the same dilemmas and challenges with ccRCC with respect to prior use of ICIs. Furthermore, while systemic therapy rates are increasing contrary to those of cytoreductive nephrectomy alone or in combination with systemic therapy in non-ccRCC, the latter is associated with lower overall mortality in appropriately selected patients (98).

## Future Perspective

How the underlying tumor biology may determine responses and resistance after initial therapy remains poorly understood. PD-L1 immunohistochemical expression has shown promise; however, variations in various assays along with use of different cutoffs for positivity are challenging. Several other candidate biomarkers were tested including tumor mutational burden, gene expression signatures, single gene mutations, human endogenous retroviruses, the gastrointestinal microbiome, and peripheral blood laboratory markers (99).

Recent gene expression analysis from the largest randomized trials in the first-line setting, that is, CheckMate 214 support a role for the inflammation status of the tumor microenvironment and PFS with ipilimumab plus nivolumab combination (100). A single-cell transcriptomic analysis of cancer and immune cells from metastatic RCC patients before or after ICI therapy revealed two subpopulations differing in angiogenic signaling and upregulation of immunosuppressive programs associated with PBRM1 mutation (101).

Appropriate selection of next-line therapies in metastatic ccRCC in the current era of ICI-ICI and ICI-VEGFR TKI combinations is challenging and there is a paucity of biomarkers to inform clinical decisions. While there is an urgent need for randomized comparative trials in this setting, given the heterogeneity of treatment responses to immunotherapy, it is unlikely that machine learning will identify a unifying transcriptional signature predictive of ORR (102–104). The BIONIKK study is the first prospective biomarker-driven phase 2 randomized trial in mRCC that will be using a 35-gene signature which reflects intrinsic disease biology to optimize selection between ICI (single or double) and VEGF TKIs in treatment-naïve patients (NCT02960906).

## Conclusion

Until ongoing clinical and translational investigations lead to the adoption of a composite panel of predictive biomarkers, the depth and duration of responses as well as clinical characteristics of the tumor and host will continue to guide next steps within this complex landscape.
